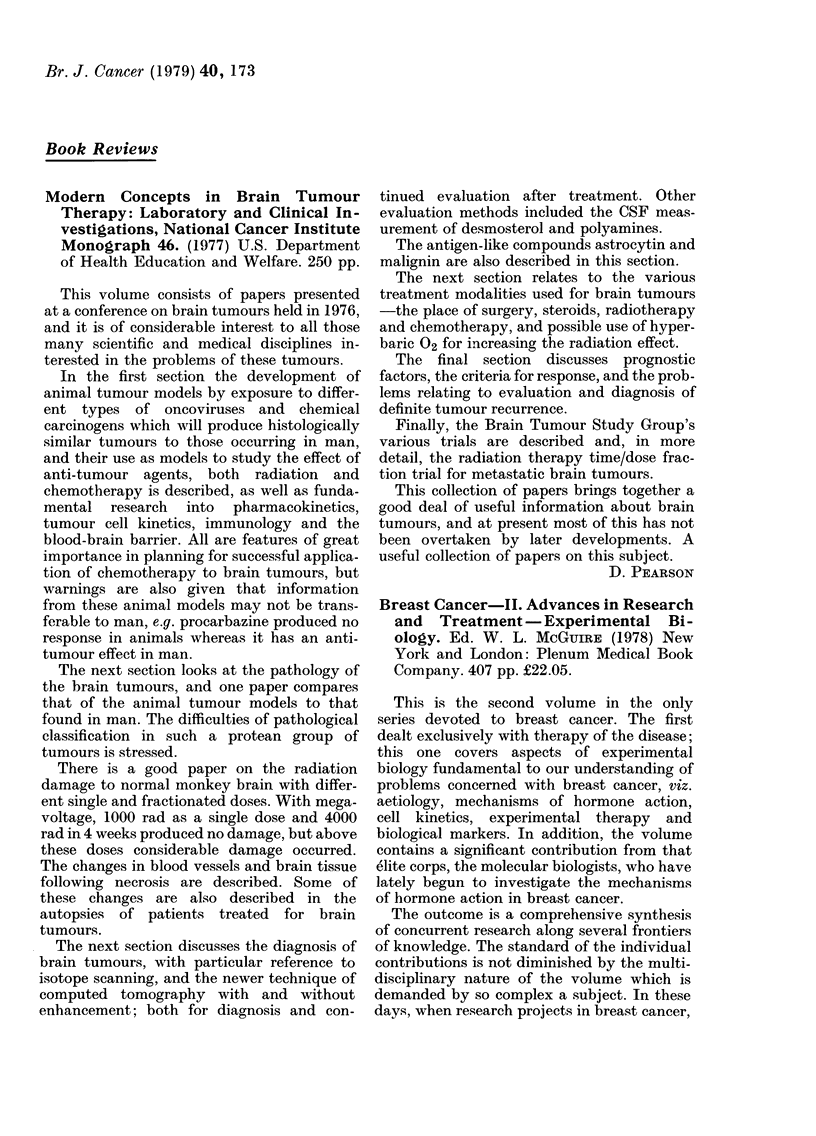# Modern Concepts in Brain Tumour Therapy: Laboratory and Clinical Investigations, National Cancer Institute Monograph 46

**Published:** 1979-07

**Authors:** D. Pearson


*Br. J. Cancer* (1979) **40**, 173

### *Book Reviews*

**Modern Concepts in Brain Tumour Therapy: Laboratory and Clinical Investigations, National Cancer Institute Monograph 46.** (1977) U.S. Department of Health Education and Welfare. 250 pp.

This volume consists of papers presented at a conference on brain tumours held in 1976, and it is of considerable interest to all those many scientific and medical disciplines interested in the problems of these tumours.

In the first section the development of animal tumour models by exposure to different types of oncoviruses and chemical carcinogens which will produce histologically similar tumours to those occurring in man, and their use as models to study the effect of anti-tumour agents, both radiation and chemotherapy is described, as well as fundamental research into pharmacokinetics, tumour cell kinetics, immunology and the blood-brain barrier. All are features of great importance in planning for successful application of chemotherapy to brain tumours, but warnings are also given that information from these animal models may not be transferable to man, *e.g.* procarbazine produced no response in animals whereas it has an anti-tumour effect in man.

The next section looks at the pathology of the brain tumours, and one paper compares that of the animal tumour models to that found in man. The difficulties of pathological classification in such a protean group of tumours is stressed.

There is a good paper on the radiation damage to normal monkey brain with different single and fractionated doses. With megavoltage, 1000 rad as a single dose and 4000 rad in 4 weeks produced no damage, but above these doses considerable damage occurred. The changes in blood vessels and brain tissue following necrosis are described. Some of these changes are also described in the autopsies of patients treated for brain tumours.

The next section discusses the diagnosis of brain tumours, with particular reference to isotope scanning, and the newer technique of computed tomography with and without enhancement; both for diagnosis and continued evaluation after treatment. Other evaluation methods included the CSF measurement of desmosterol and polyamines.

The antigen-like compounds astrocytin and malignin are also described in this section.

The next section relates to the various treatment modalities used for brain tumours —the place of surgery, steroids, radiotherapy and chemotherapy, and possible use of hyperbaric $O_2$ for increasing the radiation effect.

The final section discusses prognostic factors, the criteria for response, and the problems relating to evaluation and diagnosis of definite tumour recurrence.

Finally, the Brain Tumour Study Group's various trials are described and, in more detail, the radiation therapy time/dose fraction trial for metastatic brain tumours.

This collection of papers brings together a good deal of useful information about brain tumours, and at present most of this has not been overtaken by later developments. A useful collection of papers on this subject.

D. PEARSON